# An Unexpected Giant: A Case of Massive Paratesticular Extra-mammary Myofibroblastoma

**DOI:** 10.7759/cureus.66856

**Published:** 2024-08-14

**Authors:** Rao E Hassan, Muhammad Raza, Samah Badr Hamad, Karthiga Vasudevan, Johar Abbas

**Affiliations:** 1 Orthopaedics and Trauma, Khyber Teaching Hospital Medical Teaching Institute (MTI), Peshawar, PAK; 2 General Surgery, Ayub Medical College, Abbottabad, PAK; 3 Basic Sciences, University of Khartoum, Khartoum, SDN; 4 General Medicine, Yerevan State Medical University, Yerevan, ARM

**Keywords:** benign tumor, surgical case reports, soft-tissue sarcoma, painless scrotal mass, immunohistochemistry (ihc), open surgical excision, scrotal mass, giant tumor, paratesticular mass, mammary-type myofibroblastoma

## Abstract

Mammary-type myofibroblastomas (MFBs) are benign spindle cell tumors, typically presenting in common locations such as the breast, abdomen, and inguinal region. We present a case of a 66-year-old male with a four-year history of painless scrotal swelling. The preoperative diagnosis was challenging, with an initial suspicion of soft tissue sarcoma. A complete surgical excision was performed, revealing a well-circumscribed, encapsulated mass. The tumor measured 30 x 20 x 14 cm, weighing 5.28 kg. Histopathology confirmed an MFB. This exceptionally large paratesticular MFB emphasizes the diagnostic difficulty of such tumors. Surgical resection remains the treatment of choice, with an excellent prognosis. This case highlights the importance of considering MFB in the differential diagnosis of scrotal masses, even with atypical presentations.

## Introduction

Mammary-type myofibroblastomas (MFBs) are benign spindle cell tumors composed of fibroblasts and myofibroblasts [1]. They are commonly found in various locations, such as the breast, abdomen, chest wall, axilla, inguinal/groin region, pelvis, trunk, and extremities, with MFBs in the inguinal/groin region being the most frequently reported [2]. These tumors lack malignant potential, and the chances of recurrence are minimal [3].

We present a case of an exceptionally large paratesticular extra-mammary MFB in a 66-year-old patient, measuring a remarkable 30 x 20 x 14 cm and weighing 5.28 kg. The tumor was successfully surgically removed, and the postoperative period was uneventful, with no recurrence observed during subsequent outpatient checkups. This case report underscores the importance of considering MFB as a potential diagnosis when evaluating scrotal masses. It highlights that these tumors can attain massive sizes, posing a diagnostic challenge.

## Case presentation

**Figure 1:**
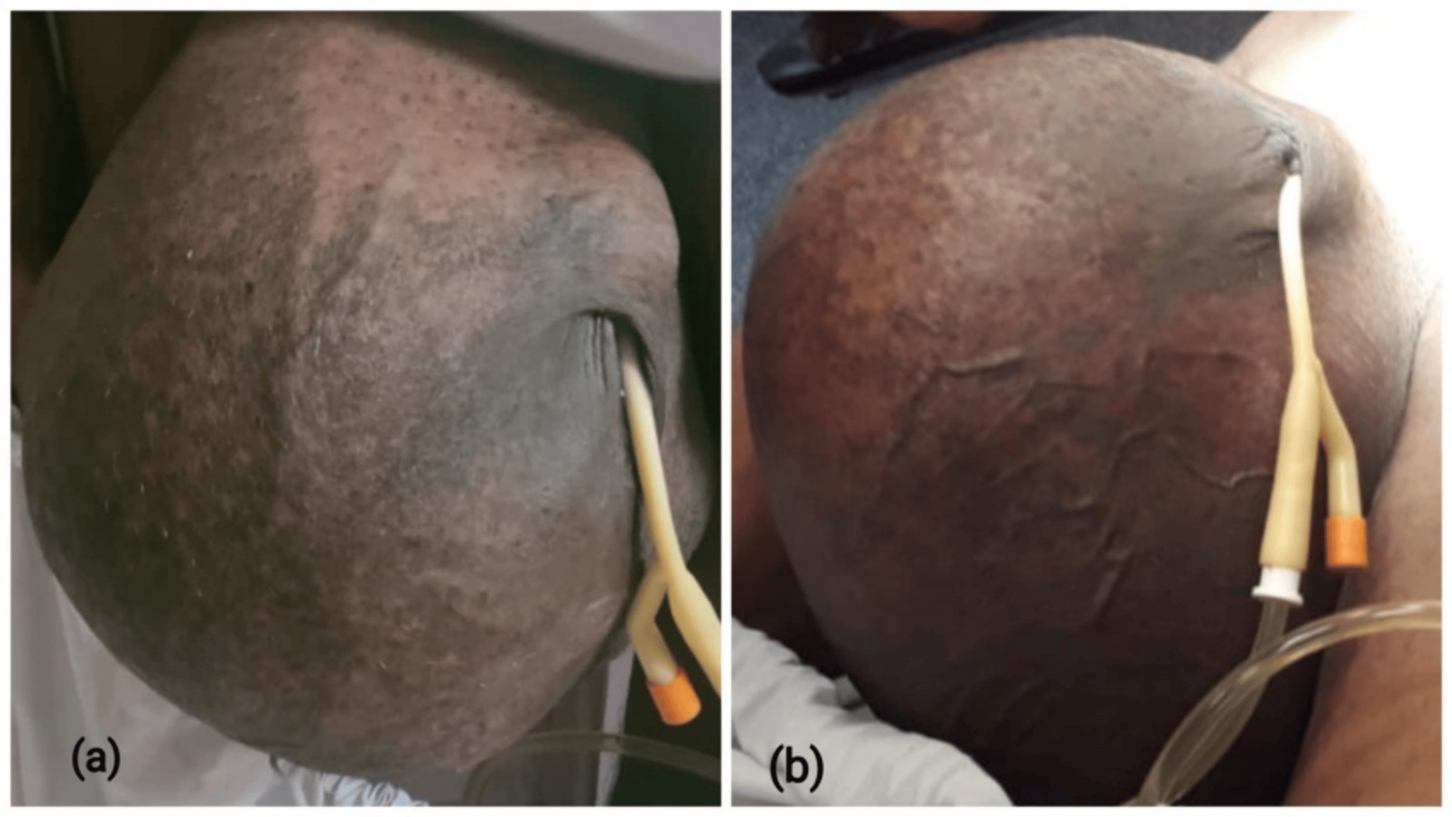
The preoperative photograph of the right paratesticular swelling, showing gains in the right-sided testicular swelling (a), which has pulled the skin over the penis (a, b)

A 66-year-old male presented with a sizable, painless swelling in the right scrotal region persisting for four years. The swelling progressively increased, causing discomfort and urinary issues as the tumor's weight pulled the skin over the penis (Figure 1). On examination, the right hemi-scrotum appeared significantly enlarged, with no skin changes except for the scrotal skin covering the penis entirely due to gravitational pull. The mass was firm and non-tender, and it was possible to get above the swelling. The right and left testicles were palpated discretely and were freely mobile, separate from the swelling. Transillumination revealed no abnormalities, and there was no inguinal or local lymphadenopathy.

The ultrasonography results suggested an inguinal hernia with omental protrusion into the scrotum, inconsistent with the physical examination. Consequently, a clinical impression of soft tissue sarcoma was made, leading to surgical exploration and excision from the right hemi-scrotum.

**Figure 2:**
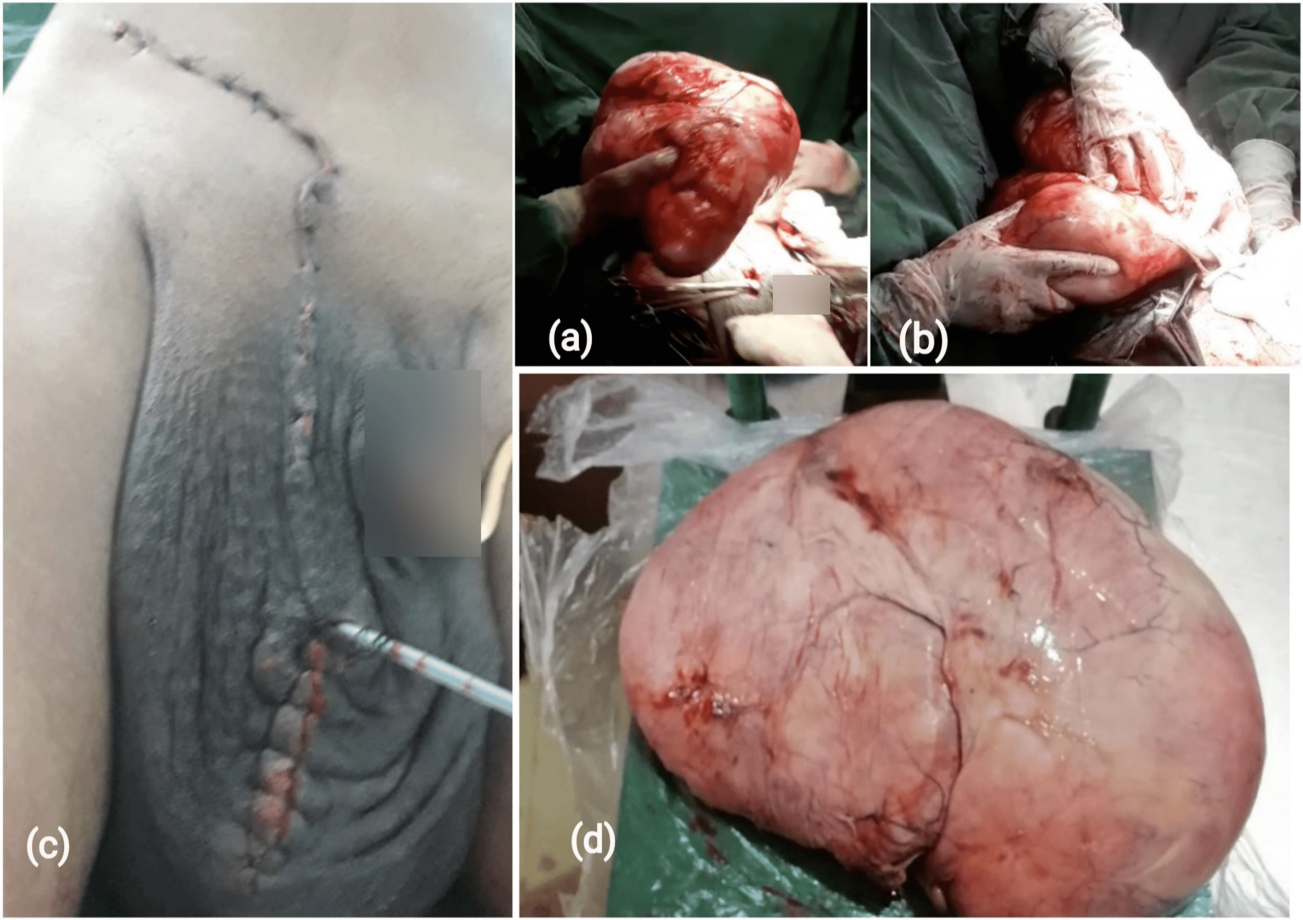
Intraoperative photographs demonstrating the size of the tumor (a, b); an immediate postoperative image showing the immediate cosmetic improvement following removal (c); and gross pathology findings (d)

During the procedure, an inguinoscrotal incision on the right side revealed a large encapsulated mass, well-circumscribed inside the scrotal sac, separate from the spermatic cord and right testicle. It was encapsulated and well-demarcated from the underlying muscles, with no invasion. The mass, measuring 30 x 20 x 14 cm and weighing 5.28 kg (Figure 2), was completely excised without leaving any remnants.

The surgery was uneventful, and the patient experienced no postoperative complications. At the three-week outpatient follow-up, the wound had healed well, and no recurrence was observed at the three-month and six-month follow-ups.

**Figure 3:**
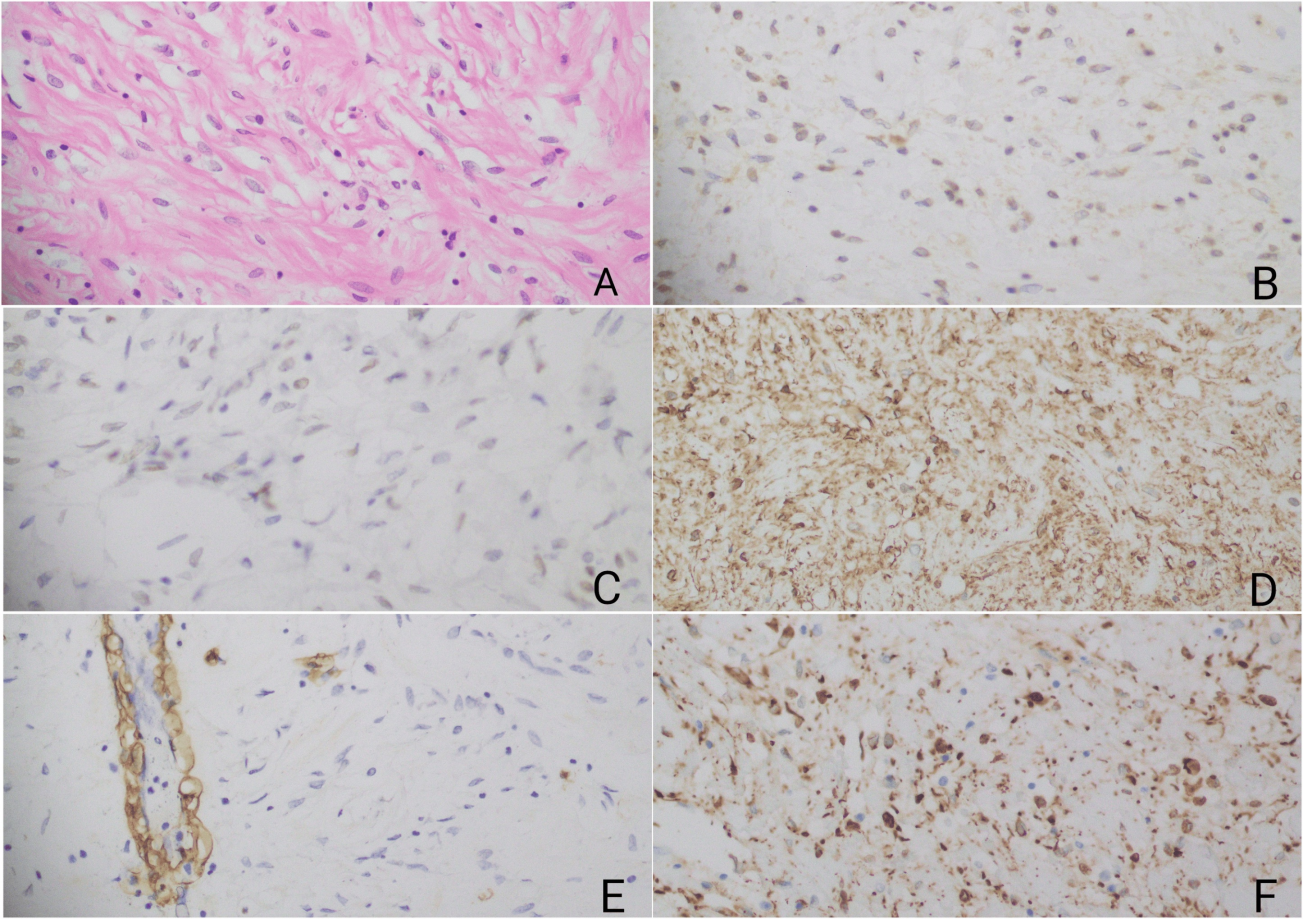
Microscopic findings of the myofibroblastoma (A) The mass consists of disorganized, variably sized fascicles of bland spindle cells. The immunohistochemical staining reveals weak positivity for ER (B), negativity for SOX10 (C), positivity for CD34 (D), positivity for SMA in vessels (E), and positivity for desmin (F) ER: estrogen receptor; SOX10: SRY-Box transcription factor 10; CD34: cluster of differentiation 34; SMA: smooth muscle actin

The histopathologic examination revealed a lesion composed of bland spindle cells with abundant collagen, along with fat cells and a few blood vessels. The immunohistochemical staining showed positivity for CD34 and desmin, weak positivity for ER, and negativity for SOX10. Smooth muscle actin (SMA) stain was also positive in the vessels (Figure 3). The histopathological diagnosis confirmed an MFB.

## Discussion

In 2020, the World Health Organization classified mammary-type MFB tumors and MFB tumors (extramammary myofibroblastic tumors) as distinct forms within the category of fibroblastic and myofibroblastic tumors [3].

First reported in 1987, MFB is a benign soft tissue tumor of the breast, characterized by well-defined borders and uniform spindle cells. Although entirely benign, the preferred treatment is surgical excision, considered a definitive cure [4]. Diagnosis involves immunohistochemistry, typically showing positivity for CD34 and desmin, along with variable staining for SMA [5]. Occurrence outside the breast is rare but closely resembles its mammary counterpart, appearing in locations beyond the mammary area [6]. A comprehensive study of 20 documented cases revealed mammary-type MFB predominantly in individuals with an advanced mean age of 52.5 years, ranging from 35 to 85 years, with a marginal male-to-female ratio of 1:0.7 [7]. The underlying rationale for the ectopic localization remains inconclusive but commonly originates contiguous to the embryonic milk line, an anatomic ridge extending from the mid-axilla to the medial groin during embryogenesis, with morphologic overlap with spindle cell lipoma [8].

In most cases, sizes typically range from 1 cm to 22 cm, with a mean size of 6.3 cm [2]. However, our unique case distinguished itself with an unusually large size of 30 cm, marking it as one of the largest paratesticular MFBs ever excised. Diagnosis is made by histopathology and staining with markers such as desmin, Rb, SMA, and CD34 [5,8]. In our specific case, the histopathological assessment revealed a lesion comprising elongated spindle cells with abundant collagen, a few blood vessels, and positive staining for SMA, confirming the diagnosis of MFB.

Treatment typically requires complete surgical removal, particularly when the mass causes noticeable effects. In cases with small and asymptomatic masses, close observation may be considered [9]. However, in our case, the gradual increase in mass size over the years resulted in discomfort and urinary complications due to the tumor's gravitational effect on the penile skin, necessitating surgical excision.

Surgeons are advised to thoroughly investigate any inguinal mass, considering potential differential diagnoses such as spindle cell lipoma, inguinal hernia, teratoma, spindle cell carcinoma, and angiomyofibroblastomas. Due to the varied nature of these conditions, a surgical approach and subsequent biopsy should be undertaken for an accurate diagnosis [9].

## Conclusions

This case report highlights the successful surgical intervention for an exceptionally large paratesticular extra-mammary MFB in a 66-year-old patient. The tumor, measuring 30 x 20 x 14 cm and weighing 5.28 kg, was surgically removed with an uneventful postoperative period and no recurrence during follow-ups. This case underscores the diagnostic challenge posed by the massive size of MFBs and emphasizes the importance of considering them in the evaluation of scrotal masses along with other differentials.
